# Effect of Craniofacial Morphology on Pharyngeal Airway Volume Measured Using Cone-Beam Computed Tomography (CBCT)—A Retrospective Pilot Study

**DOI:** 10.3390/ijerph18095040

**Published:** 2021-05-10

**Authors:** Rohan Diwakar, Anuraj Singh Kochhar, Harshita Gupta, Harneet Kaur, Maninder Singh Sidhu, Helen Skountrianos, Gurkeerat Singh, Michele Tepedino

**Affiliations:** 1 Department of Orthodontics and Dentofacial Orthopaedics, PDM Dental College and Research Institute, Bahadurgarh, Haryana 124507, India; rohandiwakar@yahoo.in; 2 Former Consultant Orthodontist Max Hospital Gurgaon, Haryana 122001, India; anuraj_kochhar@yahoo.co.in; 3 Department of Orthodontics and Dentofacial Orthopaedics, Sudha Rustagi College of Dental Sciences and Research, Faridabad, Haryana 121002, India; harshita.ortho@gmail.com (H.G.); drgurkeeratsingh@gmail.com (G.S.); 4 Department of Orthodontics and Dentofacial Orthopaedics, Faculty of Dentistry, Jamia Millia Islamia, New Delhi 110025, India; 5 Department of Orthodontics, Faculty of Dental Sciences, SGT University Gurugram, Haryana 122505, India; deanresearch@sgtuniversity.org; 6 Private Practice, Orthodontic Excellence, Puyallup, WA 98373, USA; h.skountrianos@smilewithbraces.com; 7 Department of Biotechnological and Applied Clinical Sciences, University of L’Aquila, Viale S. Salvatore, Edificio Delta 6, 67100 L’Aquila, Italy; m.tepedino@hotmail.it

**Keywords:** pharynx, airway, cone-beam computed tomography, cephalometry, craniofacial morphology

## Abstract

Background: The present study aimed to determine the correlation between pharyngeal airway volume and craniofacial morphology through cone-beam computed tomography (CBCT). Additionally, the study analyzed the influence of gender on pharyngeal airway volume. (2) Methods: 80 CBCT scans of 40 male and 40 female patients (mean age: 15.38 + 1.10 years) fulfilling the eligibility criteria were included. CBCT scans were evaluated for pharyngeal airway volume using the In Vivo Dental 5.1 software. Additionally, CBCT-derived lateral cephalograms were used to assess various craniofacial morphology parameters. To examine the influences of gender on airway volume, T-test was carried out. Correlation between airway volume and craniofacial parameters were measured using Pearson correlation followed by regression analysis. The value of *p* < 0.05 was considered statistically significant. Results: The mean airway volume was significantly greater in males than in females. A statistically significant negative correlation was found between maxillary plane inclination and pharyngeal airway volume. In contrast, a positive correlation was observed between mandibular length and lower molar inclination with oropharyngeal and total pharyngeal airway volume. Females showed a statistically significant positive correlation between the pharyngeal airway volume and sagittal position of maxilla and mandible; they also showed a negative correlation between oropharyngeal airway volume and the mandibular plane angle. Conclusions: Overall, the pharyngeal airway space differs significantly between males and females. Craniofacial morphology does have a significant effect on the pharyngeal airway, especially on the oropharyngeal airway volume.

## 1. Introduction

The upper airway has been associated with craniofacial growth. Changes in the upper airway’s normal function during the active period of facial growth could potentially influence craniofacial development [1]. Altered craniofacial morphology, such as mandibular retrognathism, short mandibular body, downward and backward rotation of the mandible, increased upper or lower face heights, the low position of the hyoid bone, increased tongue volume, enlarged palatine or adenoid tissue, and soft palate pathology are suggestive of reduced pharyngeal airway space volume [2,3]. It seems reasonable that the link between respiratory pattern and the development of malocclusion could be related to the soft tissue pressure against the dentition, which might affect the amount of tooth eruption, dental arch form and the direction of mandibular and maxillary growth [4,5,6]. The majority of studies analyzed craniofacial morphology and pharyngeal airway with 2D radiographic records such as lateral cephalograms and photographs [7]. The main limitation of two-dimensional radiographic examination is that they can provide linear measurements of the airway space but cannot represent the depth of anatomic structures and not appraise any airflow turbulence [7,8]. The use of three-dimensional radiographic records obtained from cone-beam computed tomography (CBCT) is justified in various clinical situation where detailed information about the spatial relationship of anatomic structures is needed. Three-dimensional (3D) changes in shape and position over time can be evaluated through superimpositions of sequential CBCT volumes [9]. Cone-beam computed tomography scans can be segmented in order to obtain an accurate definition of the airway volume [10,11]. Various studies have confirmed the accuracy of volumetric measurements of the airway with CBCT, yet maintaining a lower level of radiation dose than conventional computed tomography [11,12]. Since the slices in CBCT are very thin, 3D reconstruction of data allows for a clear visualization of morphology of deeper craniofacial structures. The novel CBCT machines provide increased precision, associated with a reduced dose of ionizing radiations [13]. The information obtained with the CBCT examination may influence clinical decision-making regarding the choice of treatment for growing patients with the decreased pharyngeal airway [14].

Though there is a deluge of data available on current methods to assess craniofacial morphology and its relationship with the airway space, there is a paucity of data comparing influence of gender as well as the diversity of parameters such as facial height, growth pattern, mandibular morphology, maxilla position and dentition all together. Therefore present study was undertaken to analyze the correlation between pharyngeal airway volume and the aforementioned parameters of craniofacial morphology.

## 2. Methods

In this retrospective epidemiological study, orthodontic treatment records of 150 adolescents from the Department of Orthodontics and Dentofacial Orthopaedics, SGT University (Haryana, India), were examined. Among them, 80 healthy North Indian subjects (40 boys and 40 girls mean age 15.38 + 1.10, who met the inclusion criteria) were selected. The following criteria was taken into consideration while selecting the records: no previous history of orthodontic treatment/orthognathic surgery; no facial clefts or other craniofacial anomalies; no history of medical compromise with obstructive sleep apnea; teeth in complete intercuspation, and; availability of CBCT records for orthodontic diagnosis with full field of view images.

**Figure 1:**
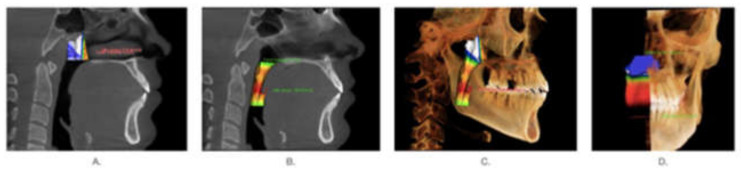
Nasopharyngeal airway segmented from lateral head films. (**A**): nasopharyngeal airway volume. (**B**): Oropharyngeal airway volume. (**C**): total airway volume. (**D**): sectional image of total airway volume.

The method of carrying out the X-ray examination and image acquisition is provided in Appendix A [15]. The following anatomic structures were identified on the lateral head film as boundaries of the nasopharyngeal airway: (1) the axial vertical plane passing through the posterior nasal spine (PNS), (2) the plane perpendicular to the axial sagittal plane from PNS extending to the superior aspect of the pterygomaxillary fissure, and (3) the soft-tissue contour of the posterior pharyngeal wall extending from the ideal of the pterygomaxillary fissure inferiorly to the axial reconstruction plane. The same planes were transferred into the 3D scan to measure airway volume over the same anatomic boundaries. The nasopharyngeal volume was defined as the pharynx volume between the palatal plane (ANS-PNS) and a line perpendicular to the palatal plane drawn from the PNS. (Figure 1A) The oropharyngeal volume was defined as the pharynx volume between the palatal plane (ANS-PNS) and the plane parallel to the palatal plane that passes from the most anterior inferior point of the second cervical vertebrae. (Figure 1B) The total pharyngeal airway volume was calculated by summing up the oropharyngeal and nasopharyngeal airway (Figure 1C).

**Figure 2:**
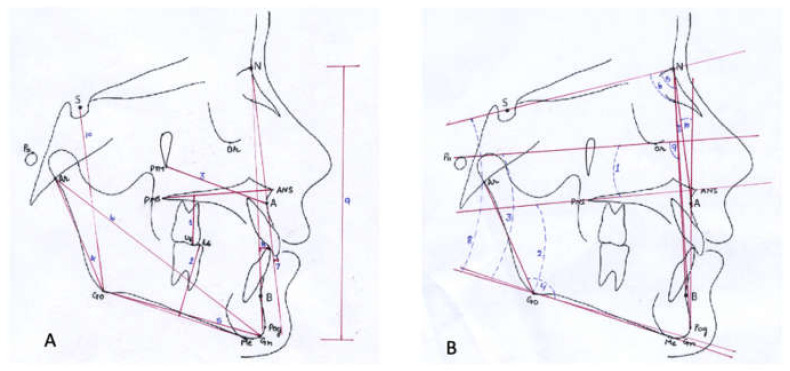
Cephalometric parameters for craniofacial morphology. (**A**): linear measurements. (**B**): angular measurements.

From the CBCT scans, lateral cephalograms were generated using In Vivo Anatomage software (Anatomage, anatomy imaging software, San Jose, CA, USA) and were further transferred to the Nemoceph software (Visiodent, Saint-Denis, France) to trace and measure craniofacial morphology (Figure 2A,B, Table 1 and Table 2). The craniofacial morphology was assessed under the following categories- (1) facial profile: facial angle (N-A:A-Pog) and facial convexity (N-A-B); (2) facial height: anterior facial height (N-Me) and posterior facial height (S-Gn); (3) mandibular morphology: total mandibular length (Ar-Gn), ramus length (Ar-Go) and mandibular body (Go-Gn); (4) maxilla: Maxillary position (Ar-Ptm) and maxillary inclination (FHP-PP); (5) growth pattern: Jarabak ratio (PFH/AFHX100), Basal plane angle (PP-MP), Mandibular plane angles (FHP-MP, and SN-MP); (6) sagittal pattern: SNA angle, SNB angle and ANB angle, (7) dentition: Upper incisor to nasion-point A plane (U1-NA), lower incisor to nasion-point B plane (L1-NB), upper first molar to palatal plane (U6-PP) and lower first molar to mandibular plane (L6-MP).

To test the inter-rater reliability of linear and volumetric measurements, the same procedures were performed by two different examiners, using the same setting.

### Statistics

Statistical analysis was performed using Statistical Package for Social Sciences (SPSS) for Windows, version 22.0 (SPSS Inc.; Chicago, IL, USA). Descriptive statistics for the various cephalometric parameters as well as pharyngeal airway volume were performed. Kolmogorov–Smirnov test analysis revealed that data was normally distributed; hence, parametric tests were applied. The difference between two groups was determined using *T*-test to examine the influences of gender on airway volume. Correlation between airway volume and craniofacial parameters, Pearson correlation and regression analysis were adopted. The level of significance was set at *p* < 0.05. To test inter-rater reliability, an interclass correlation coefficient (I.C.C.) test and weighted kappa test were calculated.

## 3. Results

The κ- value was 0.83, showing an excellent relationship to record the parameters among the examiners. Descriptive statistics were performed for both groups: males and females (Table 3 and Table 4).

The paired T-test revealed that the mean airway volume was significantly greater in males than females. (Table 4) A statistically significant negative correlation between the airway volume and plane inclination, as well as a positive correlation between oropharyngeal and total pharyngeal airway volume and mandibular length and lower molar inclination were found. (Table 5) When compared to the male counterparts, females subjects showed a statistically significant positive correlation between the nasopharyngeal airway volumes and sagittal positioning of maxilla and mandible and a negative correlation for oropharyngeal airway with the growth pattern as reflected from the mandibular plane. (FHMP) Overall, the oropharyngeal airway volume negatively correlated with the vertical craniofacial morphology and maxillary plane inclination. A positive correlation between mandibular morphology, maxilla position, sagittal pattern and dental inclination was also found. However, the results were not statistically significant.

## 4. Discussion

In the present study, airway volume was correlated with craniofacial parameters, thus emphasizing the relationship between form and function. Although it is often difficult to establish a cause effect relationship between upper airway morphology and a specific craniofacial malformations, it is demonstrated that an altered maxillo-mandibular can be associated with narrow or obstructed pharyngeal airway space, which can represent a risk factor for the Obstructive Sleep Apnoea Syndrome (OSAS). It has been known that untreated OSAS can result in serious morbidity and mortality [16]. Therefore, to our knowledge, the present 3D study assessed the relationship between pharyngeal dimensions and craniofacial morphology in both males and females. In agreement with several studies, the study results suggested that mean airway volume was significantly higher in males than females [16,17]. It has been found that irrespective of age and height, there is a difference in males and females’ pulmonary physiology. Females have airway ~30% smaller than males. The most important consequence is females have a smaller maximal flow-volume loop. Therefore, their capacity to generate increased ventilation during exercise is smaller with respect to men [17]. 

Compared to males, females showed a statistically significant positive correlation between the pharyngeal airway volumes and sagittal positioning of maxilla and mandible and negative correlation with the growth pattern as reflected from the mandibular plane angle. Similar findings were observed by Di Francesco et al. in a prospective study of 77 girls and boys. They found that sleep apnea appeared to be more severe in boys than in girls; moreover, craniofacial characteristics, such as dolichocephaly, mandibular plane, and facial depth were correlated with sleep apnea in boys [17,18]. However, Ceylan and Oktay demonstrated that the pharyngeal structures were not affected by changes in the ANB angle [19]. 

In our study, the oropharyngeal airway was directly proportional to the mandibular length (Ar-Gn and Go-Gn). Moreover, the mandibular length (Ar-Gn) was significantly correlated with the total pharyngeal airway space. These results are in agreement with the study of Trenouth and Timms, who found an association between the airway size and mandibular length. It can be hypothesized that the mandibular length could influence the distance between the airway and mandible [20]. This finding is in agreement with Muto et al., who assessed the relationship between craniofacial characteristics and the size of the pharyngeal airway space in a group of dental students through lateral head films [21]. This finding is also in agreement with the result of Solow et al., in which the pharyngeal airway was correlated with the mandibular length, measured as the linear distance between pogonion and condylion [22]. One possible reason could be that as the mandible lengthens, the genioglossus and geniohyoid muscles’ attachments move forward away from the oropharynx and thus increase the oropharyngeal airway. Many studies have addressed that mandibular retrognathism or backward rotation can induce a retrodisplacement of the tongue position and hyoid bone, leading to a concomitant decrease in the upper airway volume [23,24,25].

In the present study, the length of the maxilla (Ar-Ptm) was significantly affecting the pharyngeal airway in both groups. This finding is in agreement with Solow et al., who took cephalometric radiographs and rhinomanometry recordings for a group of young children. Solow et al. found no significant association between the pharyngeal airway and maxillary size, maxillary prognathism or maxillary inclination [22].

In the present study, maxilla’s sagittal position seems to have mildly influence on the nasopharynx and total volume of pharyngeal airway space. SNA has a positive correlation with nasopharyngeal space and entire pharyngeal airway space, while SNB has been found to influence only the nasopharyngeal space. The review of Gungor concluded that maxillary morphological differences can be detected among patients with airway problems. In the sagittal plane, shorter maxillary length, proclined maxillary incisors, increased soft palate length and thickness were noted. In the transverse plane, patients with airway problems presented narrow, V-shaped maxillary arch, and a high palatal vault [26,27,28]. The authors reported that a constricted nasopharyngeal airway is associated with a retruded mandible as well as a retruded maxilla [28].

According to Xu et al., pharyngeal airway volume was smaller in cases where the condyle was anteriorly placed compared to the condyle’s centric and posterior position in class II subjects [29]. The study was based on different condylar positions, and the subjects were divided accordingly along with patients age and sex. There was no significant difference in patient age or sex distribution, ANB, SNB, Wits, or MPFH between the groups. In contrast, patients with an adequate airway space can still maintain good airway conditions, even if the mandible and condyle are posteriorly positioned, which may explain the finding that the volume and area of the pharyngeal airway space were larger in the posterior group and was significantly smaller in the centric and anterior groups.

In a study conducted by Joy et al. [30] Pliska et al. [31] and Zhang et al. [32], the relationship between extraction of premolars and changes in airway dimensions were noted during orthodontic treatment. They stated that dental extractions affect dental features such as upper and lower incisor position and inclinations and intermolar width. However, there was no evidence that extraction changed sagittal and transverse distances or minimal cross-sectional area or volume in the nasopharyngeal, retropalatal, or retroglossal regions. There was no evidence that changes in the measured skeletal or dental features had an indirect effect on airway features.

### 4.1. Clinical Applications of the Study

The present study highlights the pharyngeal airway volume was significantly correlated with craniofacial parameters, thus highlighting the association between form and function. There is also a strong interaction between craniofacial morphology, obesity, hypertension, ageing and breathing disorders such as OSAS [33,34,35]. Clinically, most of the essential parameters that are usually modified or modulated during orthodontic/orthopaedic/orthognathic treatment have been selected so as to get a better understanding if any relation exists directly or indirectly with the airway volumes. If a patient reports with symptoms like mouth breathing/snoring/obstructive sleep apnoea that are associated with narrow pharyngeal airway, treatment should be directed towards eliminating the underlying etiology based on correction of the skeletal morphology as well.

### 4.2. Strengths and Limitations of the Study

In our study, we have included majority of the parameters of craniofacial morphology such as facial height, growth pattern, mandibular morphology, maxilla position and dentition, have never been assessed together in previous studies. Also, since the medically compromised subjects were excluded in our study, a direct influence of craniofacial morphology on the pharyngeal airway cannot be contemplated. The pathways in which variation in the airflow can influence growth and development have not been completely illuminated [36,37]. As a main limitation, clinical examination of the study sample was not possible, due to the retrospective nature of the present study. Therefore, the analysis was based on CBCT records, and it was not possible to correlate the radiographic findings with any clinical sign and/or symptom of airway obstruction. Therefore, we would consider this study a pilot study for future research.

## 5. Conclusions

In the present study, the pharyngeal airway volume was significantly correlated with craniofacial parameters, thus highlighting the association between form and function. Overall, the pharyngeal airway space also differed significantly between the sexes.

## Figures and Tables

**Table 1:** Various landmarks used in the cephalometric tracing.

Landmark	
Sella (S)	Center of pituitary fossa
Nasion (N)	Anterior most point of frontonasal suture seen as triangular projection with irregular margins
Orbitale (O)	Inferior-most point on lower margin of rim of orbit
Porion (Po)	Superior-most point on external auditory meatus
Pterygomaxillary Fissure (Ptm)	Inferior-most point of the inverted pear shaped radiolucency seen in posterior maxillary region
Articulare (Ar)	Superior-most point of condyle of mandible
Anterior Nasal Spine (ANS)	Most anterior point on the anterior projection of roof of maxilla or floor of nasal cavity
Posterior Nasal Spine (PNS)	Most posterior point on the posterior projection of roof of maxilla
Menton (Me)	Most inferior point of mandibular symphysis
Pogonion (Pog)	Most anterior point on mandibular symphysis
Gnathion (Gn)	Most antero-inferior point of mandibular symphysis present between Pog and Me

**Table 2:** Various parameters used in the cephalometric tracing.

Parameter	
U6-PP	Linear measurement formed between the mesiobuccal cusp of the upper molar and palatal plane along the long axis of the molar
L6-MP	Linear distance between the mesiobuccal cusp of the lower molar and the mandibular plane along the long axis of the molar
A-Ptm	Linear distance between point A and Ptm
Ar-Go	Linear distance between point Articulare and Gonion
Go-Gn	Linear distance between point Gonion and Gnathion
Ar-Gn	Linear distance between Articulare and Gnathion
Upper incisor to NA	Linear distance between the line joining NA and the incisal tip of upper central incisor
Lower incisor to NB	Linear distance between the line joining NB and the incisal tip of lower central incisor
Anterior face height	Linear distance between Nasion and Menton
Posterior face height	Linear distance between Sella and Gonion
FH-PP	Angle between Frankfort plane and Palatal plane
PP-MP	Angle between Palatal plane and Mandibular plane
FH-MP	Angle between Frankfort plane and Mandibular plane
Gonial angle	Angle formed between Articulare, Gonion and Menton
SNA	Angle formed between Sella, Nasion and Point A
SNB	Angle formed between Sella, Nasion and Point B
ANB	Angle formed between Point A, Nasion and Point B
SN-MP	Angle formed between line joining Sella and Nasion and the line joining Gonion and Gnathion
Facial Angle	Angle formed by intersection of line joining Nasion and Pogonion with the Frankfurt Horizontal
Facial convexity	Angle formed between line joining Nasion and Point A and the line joining Point A and Pogonion

**Table 3:** Descriptive statistics and paired t-test for comparison of various cephalometric parameters for craniofacial morphology.

Parameters	Male		Female		*t*-Test	*p*-Value
	Mean	SD	Mean	SD		
Facial Profile						
FA	90.37	4.67	88.70	5.41	2.19	0.14
FC	7.13	5.23	7.26	6.42	0.01	0.92
Facial Height						
AFH	99.86	20.01	89.33	20.89	5.30	0.02 *
PFH	69.50	14.42	60.36	14.01	8.29	0.005 *
Mandibular Morphology						
ArGn	92.97	22.73	84.02	19.45	3.58	0.06
GoGn	66.74	14.19	56.29	13.13	10.04	0.002 *
ArGo	42.26	10.46	36.57	9.03	6.78	0.01 *
Maxilla Position						
ArPtm	43.50	8.71	37.88	9.64	7.48	0.008 *
FHPP	−1.02	3.29	−0.37	3.12	0.84	0.36
Growth Pattern						
Jarabak	69.65	4.84	67.76	5.29	2.81	0.09
PPMP	23.77	6.01	26.33	7.58	2.81	0.09
FHMP	22.36	7.11	25.15	7.82	2.79	0.09
SNMP	28.94	5.29	32.11	6.33	5.91	0.02 *
Sagittal pattern						
SNA	83.97	4.55	82.32	3.26	3.48	0.07
SNB	79.78	4.79	78.32	4.03	2.18	0.14
ANB	4.19	2.08	4.01	2.89	0.11	0.75
Dentition						
U1NA	4.49	2.35	3.33	1.82	6.04	0.02 *
LINB	5.35	1.93	4.91	2.79	0.69	0.41
U6PP	20.03	4.83	18.34	5.03	2.34	0.13
L6MP	27.95	5.95	24.81	6.04	5.49	0.02 *

* Statistically significant with *p* < 0.05. SD: standard deviation; FA: facial angle; FC:facial convexity; AFH: anterior facial height; PFH: posterior facial height; ArGn: articulare to gnathion; ArGo: articulare to gonion; GoGn: gonion to gnathion; ArPtm: articulare to pterygomaxillary fissure; FHPP: frankfort horizontal plane- palatal plane; PPMP: palatal plane- mandibular plane; FHMP: frankfort horizontal plane- mandibular plane; SNMP: sella nasion plane- mandibular plane; SNA: sella-nasion-point A; SNB: sella-nasion-point B; ANB: point A-nasion-point B; U1NA: upper incisor to nasion-point A; L1NB: lower incisor to nasion-point B; U6PP: upper first molar to palatal plane; L6MP: lower first molar to mandibular plane.

**Table 4:** Descriptive statistics and Paired t-test for Comparison of Airway volume parameters among males and females.

Parameters	Males		Females	Total			*t*-Test	*p*-Value
	Mean	SD	Mean	SD	Mean	SD		
Nasopharynx	6.24	2.04	5.27	1.73	5.76	1.94	5.27	0.02 *
Oroharynx	13.76	6.81	10.47	3.94	12.11	5.77	7.01	0.01 *
Total	19.95	7.69	15.44	4.78	17.69	6.75	9.93	0.002 *

* Statistically significant with *p* < 0.05. SD: standard deviation.

**Table 5:** Pearson Rank Correlation between airway volume and craniofacial morphology.

Parameters		Male			Female			Total		
		NASO	ORO	Total	NASO	ORO	Total	NASO	ORO	Total
Facial Height										
AFH	r value	−0.06	0.12	0.087	0.033	0.118	0.111	0.049	0.177	0.170
PFH	r value	−0.04	0.04	0.021	0.098	0.209	0.181	0.100	0.178	0.175
Growth Pattern										
PPMP	r value	0.17	0.07	0.114	−0.178	−0.17	−0.09	−0.06	−0.08	−0.05
FHMP	r value	0.19	0.12	0.173	−0.081	−0.41 *	−0.28	0.009	−0.128	−0.07
SNMP	r value	0.18	0.10	0.143	−0.12	−0.23	−0.16	−0.04	−0.11	−0.08
Mandibular Morphology										
ArGn	r value	−0.02	0.22	0.189	0.083	0.210	0.183	0.076	0.262 *	0.241 *
GoGn	r value	−0.16	0.07	0.018	0.148	0.240	0.227	0.055	0.209	0.191
ArGo	r value	−0.17	−0.09	−0.14	0.134	0.270	0.240	0.030	0.103	0.090
Maxilla Position										
Ar-Ptm	r value	−0.19	0.13	0.05	0.002	0.157	0.109	−0.013	0.205	0.164
FHPP	r value	0.14	−0.07	−0.01	0.026	−0.55 *	−0.47 *	0.059	−0.25*	−0.20 *
Sagittal pattern										
SNA	r value	−0.16	0.04	−0.02	0.648 *	0.241 *	0.452 *	0.189	0.153	0.187
SNB	r value	−0.13	0.004	−0.04	0.541 *	0.084 *	0.251 *	0.185	0.076	0.114
ANB	r value	−0.05	0.08	0.038	−0.022	0.149	0.156	−0.024	0.105	0.093
Facial Profile										
FA	r value	−0.27	−0.18	−0.24	0.190	0.142	0.102	0.002	0.004	−0.03
FC	r value	−0.16	−0.05	−0.11	0.037	0.181	0.217	−0.060	0.036	0.025
Dentition										
U1NA	r value	−0.02	−0.05	−0.05	−0.058	0.081	0.016	0.038	0.070	0.067
LINB	r value	−0.04	0.19	0.150	−0.030	00.198	0.178	−0.010	0.193	0.172
U6PP	r value	0.03	−0.01	0.01	0.152	0.148	0.168	0.125	0.096	0.122
L6MP	r value	0.02	0.17	0.154	0.100	0.210	0.196	0.119	0.240 *	0.237 *

* Statistically significant with *p* < 0.05. NASO: nasopharynx; OROPH: oropharynx; FA: facial angle; FC: facial convexity; AFH: anterior facial height; PFH: posterior facial height; ArGn: articularae to gnathion; ArGo: articularae to gonion; GoGn: gonion to gnathion; ArPtm: articularae to pterygomaxillary fissure; FHPP: frankfort horizontal plane- palatal plane; PPMP: palatal plane- mandibular plane; FHMP: frankfort horizontal plane- mandibular plane; SNMP: sella nasion plane- mandibular plane; SNA: sella-nasion-point A; SNB: sella-nasion-point B; ANB.: point A-nasion-point B; U1NA: upper incisor to nasion-point A; L1NB: lower incisor to nasion-point B; U6PP: upper first molar to palatal plane; L6MP: lower first molar to mandibular plane.

## Appendix A

Cone-beam computed tomography (CBCT) scans were acquired with i-Cat. Cone Beam 3-D Dental Imaging system (i-CAT Classic, Imaging Sciences International, Hatfield, PA, USA). The image acquisition followed a standardized protocol, with the subject seated in a chair; the machine settings were as follows: 120 kV, 5 mA, 0.25 mm voxel, and scan time of 20 s. The Dicom images were imported to In Vivo Dental 5.1 (Anatomage, anatomy imaging software, San Jose, CA, USA): from the coronal view, the volume was rotated mediolaterally until the transporionic line of the data becomes horizontal and parallel to the screen outline; from axial view, the volume was rotated until the midsagittal plane of the data oriented vertically and parallel to the screen outline; from the sagittal view, the volume was rotated anteroposteriorly so that the Frankfurt plane of the data was oriented horizontally and parallel to the screen outline [15]. Using the inherent feature of the software, the images were reoriented and segmented and finally the 3D reconstruction of total pharyngeal airway volume was exported in .jpg format and the measurements were done.
